# Multimericity Amplifies the Synergy of BCR and TLR4 for B Cell Activation and Antibody Class Switching

**DOI:** 10.3389/fimmu.2022.882502

**Published:** 2022-05-19

**Authors:** Egest J. Pone, Jenny E. Hernandez-Davies, Sharon Jan, Emily Silzel, Philip L. Felgner, D. Huw Davies

**Affiliations:** Vaccine Research & Development Center, Department of Physiology & Biophysics, School of Medicine, University of California, Irvine, Irvine, CA, United States

**Keywords:** multimericity, B cells, class switching, B cell receptor (BCR), Toll-like receptor (TLR), synergy, streptavidin, biotin

## Abstract

Sustained signaling through the B cell antigen receptor (BCR) is thought to occur only when antigen(s) crosslink or disperse multiple BCR units, such as by multimeric antigens found on the surfaces of viruses or bacteria. B cell-intrinsic Toll-like receptor (TLR) signaling synergizes with the BCR to induce and shape antibody production, hallmarked by immunoglobulin (Ig) class switch recombination (CSR) of constant heavy chains from IgM/IgD to IgG, IgA or IgE isotypes, and somatic hypermutation (SHM) of variable heavy and light chains. Full B cell differentiation is essential for protective immunity, where class switched high affinity antibodies neutralize present pathogens, memory B cells are held in reserve for future encounters, and activated B cells also serve as semi-professional APCs for T cells. But the rules that fine-tune B cell differentiation remain partially understood, despite their being essential for naturally acquired immunity and for guiding vaccine development. To address this in part, we have developed a cell culture system using splenic B cells from naive mice stimulated with several biotinylated ligands and antibodies crosslinked by streptavidin reagents. In particular, biotinylated lipopolysaccharide (LPS), a Toll-like receptor 4 (TLR4) agonist, and biotinylated anti-IgM were pre-assembled (multimerized) using streptavidin, or immobilized on nanoparticles coated with streptavidin, and used to active B cells in this precisely controlled, high throughput assay. Using B cell proliferation and Ig class switching as metrics for successful B cell activation, we show that the stimuli are both synergistic and dose-dependent. Crucially, the multimerized immunoconjugates are most active over a narrow concentration range. These data suggest that multimericity is an essential requirement for B cell BCR/TLRs ligands, and clarify basic rules for B cell activation. Such studies highlight the importance in determining the choice of single vs multimeric formats of antigen and PAMP agonists during vaccine design and development.

## Introduction

Appropriate B cell responses are essential for vaccine efficacy since class switched high affinity antibodies secreted by plasma cells neutralize existing pathogen. B cell differentiation is hallmarked by CSR of constant heavy chains from IgM/IgD to IgG, IgA or IgE isotypes, as well as SHM mainly in complementarity determining regions (CDRs) of antibody variable light and heavy chain genes. Furthermore, vaccine- or infection-elicited memory B cells are held in reserve for future encounters, and activated B cells also serve as semi-professional antigen presenting cells (APCs) for T cells. Although the main signaling pathways for B cell activation and differentiation are largely known *individually* (1–4), further understanding is required regarding their particular combination and strength, which critically depend on the geometry, formulation and ratio of the corresponding eliciting ligands (5–8).

Microbes typically contain both repetitive antigens, which are recognized by the clonally-encoded BCR and T cell receptor (TCR), as well as multiple copies of several distinct types of pathogen associated molecular patterns (PAMPs), which are recognized by their germline-encoded pattern recognition receptors (PRRs). In viruses there are only a handful of protein components and typically one of them is the main, or dominant, protein antigen which serves to attach to host cells and in turn is the main focus of the antibody-mediated immune response (9). In the more complex bacteria and parasites there are hundreds of constituent proteins, but again, there usually are only a few main outer membrane protein antigens, as well as nonproteinaceous antigens, (each in many repetitive copies) that mediate binding to host cell receptors, along with numerous accessory antigens (attachment and virulence co-factors, toxins). These ‘immunodominant’ antigens are preferentially, though not exclusively, recognized by the immune system and an appropriate cellular and humoral immune response targeting them is mounted (10).

There seems to be growing consensus that repetitive, multimeric antigens elicit stronger B cell differentiation compared to soluble antigens (5, 8). Thus, vaccine preparations that maintain the native antigen conformation, density and topology are expected to be superior to those that use soluble monomeric antigen and separate adjuvants (8). In particular, nanoparticles present highly clustered and repetitive antigen(s) together with appropriate co-stimulatory agonists that enter lymphatics and collect in draining lymph nodes to stimulate robust B and T lymphocyte interactions in the nascent germinal centers (6, 8, 11). In contrast, the role that multimeric forms of PRR agonists play in B cell responses is less well known, despite vaccine adjuvants frequently employing attenuated or inactivated whole pathogens, recombinant viruses, or various carriers (lipids, polymers) which to some extent present either the original architecture of multimeric ligands or a synthetic form of clustered ligands. Likewise, investigating the sufficient and optimal combination and amount of different types of stimuli needed to elicit protective B and T cell responses has been an understudied aspect of vaccine design (12, 13).

BCR is the quintessential receptor which detects non-self antigens (and autoantigens in the abnormal case of autoimmune disease) and licenses B cells for activation and priming in an antigen-specific manner. Although the resting BCR may be distributed in random monomeric units on the plasma membrane, there is evidence that sustained BCR signaling only occurs when antigen(s) crosslink multiple BCR units (variously termed BCR nanoclusters, islands or rafts) for relatively stable duration on the order of minutes to hours or even days (14, 15). It is now thought that at least dozens of BCRs are required to assemble around antigen for sustained BCR signaling to occur (16), as first proposed in the 1970s in the “immunon model” of BCR signaling (17). The most effective antigens for engaging the BCR are thought to be those that are present in multiple copies and are arrayed in the same orientation, thereby turning the individual affinity of antigen for the antibody variable regions into a greater avidity (i.e. the overall multimeric affinity) that is physiologically competent to trigger sustained BCR signaling. This notion has been clearly described and explained by Hinton and colleagues (5). This nanoscale signalosome organization appears to be widespread in biology, as can be observed in the oligomeric organization of the T cell receptor (TCR) (18), the IL-2 receptor alpha chain (CD25) (19), several innate immune receptors collectively termed supramolecular organizing centers (SMOCs) (20) or supramolecular activation clusters (SMACs) (21). These signaling receptor units assemble in nano-scale clusters to transmit signals in an ultrasensitive and cooperative manner, i.e. having a high threshold (to discriminate against spurious noise), then rising in a sigmoidal manner before saturating (22–24). While oligomeric ligands have been studied in a variety of receptor and cell types, they are particularly suitable for research in vaccine design since, on the one hand, the BCR may require crosslinking by multiple antigen copies, and on the other hand, the clonally distributed BCR may display differential affinity for antigen variants in their single vs oligomeric forms.

Following the original immunon model (17) on the role of multimeric antigens in antibody responses *in vivo*, researchers in the field typically mimic the natural strong and sustained BCR signaling *in vitro* by using anti-IgM/anti-IgD antibodies that are immobilized either on the culture dish surface (i.e. are plate-bound), on solid beads such as microparticles and nanoparticles, or on polymers such as dextran (25, 26), all of which engage the BCR in a multivalent manner. Early reports indicated that hyper-crosslinking the BCR with anti-IgD conjugated to Ficoll or to dextran biopolymers (27), by anti-IgM conjugated to dextran (28, 29), or by biotinylated anti-IgM antibodies bridged by streptavidin (30) triggered stronger BCR signaling and B cell activation compared to the soluble anti-IgD/anti-IgM antibodies. These reports, however, did not quantify B cell differentiation exemplified by class switching after several days in culture. A subsequent important study (31) found that anti-IgD-dextran was essential to induce B cell activation and CSR to IgA in the presence of LPS or CD40 ligand and IL-4, IL-5 and TGFβ1, which has remained the standard method for inducing IgA *in vitro*. Finally, following this methodology, a recent study (32) used anti-IgD-dextran and the accessory reagents (but without TGFβ1) to induce robust CSR to IgG1 as measured by flow cytometry.

After initially sensing antigens on their surface BCR, B cells typically internalize soluble or particulate antigen so that it can be processed in endosomes containing MHCII (for antigen presentation to T cells) (33) and endosomes/phagosomes containing PRRs for detection of microbial type and co-activation (34–36). Besides mandatory expression of the BCR, B cells highly express several types of PRRs. B cells responses are heavily influenced by PRRs (7), by the BCR, by receptors for co-stimulation or help by T cell and dendritic cell (DC), and by cytokine, chemokine and other accessory receptors. Similar to their role in activating innate immune cells, PRRs and cytokine receptors provide nonredundant co-stimulatory signals for B cell growth and proliferation. The ensuing outcomes, i.e. extent of cell enlargement, proliferation, the antibody isotype expressed, other differentiation markers along the germinal center, memory or plasma cell pathway, depend on the strength and duration of the various activated receptors (37–40). Antigens and co-stimuli essentially act as growth factors, or mitogens (41) and cause lymphocytes to enter the cell cycle and divide several times. Therefore the amount and sustained presence of the mitogens are critical to sustaining the expanding lymphocyte clones, or blasts. In addition to a minimum number/density of antigens on a single particle, it is important to emphasize that full B cell activation and differentiation generally requires interaction and internalization of one or more types of PRRs and cytokine receptors. However, PRRs have been more extensively studied in innate immune cells than in lymphocytes. In macrophages and DCs, the PRR dectin-1, which detects fungal beta-glucans (42), was activated at significantly higher levels by multimeric, particulate ligands compared to their monomeric, soluble forms. Likewise, while some TLRs can detect individual monomeric ligands, they are activated to higher levels by forming nanoclusters of the same kind (homotypic) or different kinds (heterotypic) of receptors (43, 44). The cellular basis for such heightened responses is considered to be the formation of particular lipid rafts (44), recruitment and stabilization of downstream signaling mediators (43, 44), endocytosis or phagocytosis and its signaling (45), cytoplasmic phase separation into receptor and mediator rich liquid-like condensates (46), or combinations of the above.

Inspired by this previous extensive body of literature, the focus of the present report is the effect of multimeric anti-IgM and multimeric LPS on B cell activation and class switching. In our present study, we first performed an unbiased screen of BCR, PRR and cytokine receptors to measure any potential synergy in inducing B cell growth and antibody class switching. Importantly, LPS and anti-IgM/IgD stood out from over a dozen stimuli screened for their ability to activate B cells and trigger CSR. In addition, B cell stimulation assays *in vitro* revealed that crosslinking of biotinylated LPS and anti-IgM by streptavidin, or immobilized on nanoparticle coated with streptavidin, needs to be empirically ‘tuned’ in order to cause higher B cell growth and differentiation as measured by antibody class switching. Experiments with conjugates of biotinylated stimuli and either streptavidin, 8-arm polyethylene glycol (PEG) dendrimer conjugated with 8 streptavidins, or the more stable binding streptavidin variant called traptavidin, all showed similar profiles of enhancement of B cell class switching. The LPS and anti-IgM immunoconjugates had a molecular weight higher than 300 kDa as measured by molecular sieving experiments. In summary, these results suggest that presenting repetitive antigen together with co-stimulatory repetitive PRR agonists are potent inducers of B cell mobilization and antibody class switching.

## Materials and Methods

### Reagents

Antibodies and cytokines were obtained from Biolegend (San Diego, CA). *S. abortus equi* LPS (biotinylated or non-biotiylated) was obtained from Adipogen (San Diego, CA), and *E. coli* LPS was from Sigma-Aldrich (St. Louis, MO). Unlabeled or biotinylated goat anti-mouse IgM (Fab’)_2_ was from Southern Biotech (San Diego, CA). Unlabeled or biotinylated monoclonal anti-mouse IgM (clone RMM1), anti-mouse IgD (clone 11-26c), anti-mouse IL-4 (clone BVD6-24G2), and other mAbs to CD markers were from Biolegend (San Diego, CA). Phosphorothioate backbone CpG-1826 and its 3-fold repetition CpG-1826-3X were purchased from IDT (Coralville, IA). Streptavidin-coated polystyrene nanoparticles (103 nm in diameter) were from Bangs Laboratories (Fishers, IN); they are supplied as a 1% slurry and volume concentrations refer to this original concentration (divide by 100 to get the absolute concentration of the solid volume of nanoparticles present in culture). A streptavidin dendrimer consisting of 8-arm polyethylene glycol (PEG) with each arm bound covalently to a streptavidin was purchased from Creative PEGWorks (Chapel Hill, NC). ACK red blood cell lysing buffer was from Lonza (Basel, Switzerland). Centrifugal molecular filtration devices were from Vivaspin. General pipets, V-bottom polypropylene plates, tissue culture plates and accessory reagents were from Thermo Fisher Scientific (Waltham, MA) or USA Scientific (Ocala, FL).

### B Cell Cultures

C57BL/6 mice were obtained from Charles River Laboratories. Conjugates of LPS, anti-IgM, PPR agonists and CD markers were first assembled in a V-bottom polypropylene 96 well plate at room temperature (rt) for 2 hrs in a final volume of 5 to 30 μl DPBS (note that these low adsorbing plates are typically used for qPCR, but were used here for immunoconjugate assemblies). Then the assembled conjugates added to each corresponding well of cells cultured in RPMI-FBS in 96 well flat bottom tissue culture-treated plates. Typical biotinylated stimuli included the following T-independent (T-I) stimuli: BCR reagents (0.15 μg/ml biotin-anti-IgD clone 11-26c, 0.15 μg/ml biotin-anti-IgM clone RMM1), PRR agonists (0.5 μg/ml biotin-LPS from either *E. coli* or *S. abortus equi*, 1.5 μg/ml biotin-CpG-1826, 5 μg/ml biotin-CpG-1826-3X). Additional stimuli included a T-dependent (T-D) stimulus (0.5 μg/ml biotin-anti-CD40), and other CDs for comparison (0.5 μg/ml biotin-anti-CD19, 0.5 μg/ml biotin-anti-CD21, 0.5 μg/ml biotin-anti-CD38, 0.5 μg/ml biotin-anti-CD284). The above reagents were first mixed individually or in combinations as indicated in main text and figures with 1 μg/ml streptavidin (except where elsewhere titrated in various concentrations as indicated) in each well of a V-bottom 96 well plate at rt for 2 hrs. DPBS was used to bring volume for each well of these biotinylated stimuli conjugated to streptavidin to a final 20 μl, except when performing the molecular sieving experiments which required larger volumes indicated further below. The concentrations of stimuli can be converted to absolute amounts added per 250 μl V-well during the first biotin conjugation step by dividing by 4, e.g. 1 μg/ml streptavidin per cell culture means that 0.25 μg streptavidin was added to each well.

These immunoconugates were then used to stimulate single spleen cell suspensions depleted of red blood cells (1 x 10^5^ to 3 x 10^5^ cells/ml in each well) from mouse spleens in FBS-RPMI medium supplemented with 50 μM beta-mercaptoethanol (BME) and, where indicated 25 ng/ml IL-4, in flat bottom 96 well tissue culture plates in a humidified CO_2_ incubator for 3 to 4 days as shown in main text. For the majority of these cell assays the starting culture was splenocytes rather than purified B cells since a main long-term goal of the project is to evaluate vaccine adjuvants in a suitable cell culture model in order to predict their effects in immunizations *in vivo*; selected experiments comparing splenocyte starting cultures to purified B cells revealed no essential differences in final B cell growth and differentiation.

### Flow Cytometry

Single cell suspensions from the cell culture plates were transferred to round bottom plates and cells collected by centrifugation in a swing plate rotor (Beckman) at 500 g for 5 min. The cells were labelled at room temperature (rt) for 30 min with a cocktail of fluorescent Abs and reagents, typically comprising: Pacific Blue-anti-IgD, BV510-anti-CD138, BV655-anti-IgG1, BV785-anti-CD19, FITC-anti-IgG3, PE-anti-CD3, PE/Cy-anti-GL7, APC-anti-IgA, APC/Cy7-anti-IgM (all from BioLegend Inc.) and 7AAD (AnaSpec, Fremont, CA) in a final volume of 50 μl in DPBS buffer. Samples were washed with 200 μl DPBS, centrifuged as before, and resuspended in 115 μl DPBS. Next, 100 μL samples were acquired from each well of the 96 round well plates on a Novocyte 3000 flow cytometer for 50 sec, with FSC-H threshold set at 100,000, and voltage settings such that unstained cells have autofluorescence values between 10^2^-10^3^ units. Unstained and single-color stained controls were used to set up the compensation matrix.

Flow data were analyzed with FlowJo software (FlowJo LLC., Ashland, OR). Live lymphocytes were gated by appropriate forward vs side scattering, followed by singlet gating by area vs. height of forward scattering pulse, and finally followed by 7AAD^-^ gating of live (non-apoptotic and non-necrotic cells). Next, CD19^+^ (including dim or lo and bright or high) cells were gated first (at the end of 3 to 4 days of stimulated cell cultures, these typically comprised 95-100% of all cells). This gating was followed by gating for IgG1^+^ (class switched) cells, CD138^+^ CD19^lo^ (plasma cells), or PNA+ (germinal center-like B cells).

### Molecular Filtration of Immunoconjugates

Biotin-LPS, biotin-anti-IgM and biotin-anti-IL-4 in complex with IL-4 were conjugated to streptavidin, and this mixture is referred to as input. The input was centrifuged through a Vivaspin 6 ultrafiltration (molecular sieving) filter with a 300 kDa cut-off, and the supernatant and flow-though were collected. The supernatant volume was brought to be equal to the volume of flow-through by adding DPBS. Next, equal volumes of input, supernatant and flow-through were added to splenocyte cultures at 0, 0.3X, 1X, and 3X, where 1X refers to the initial concentration of each component in the input.

### Statistical Analysis

Statistical analysis was performed with Excel (Microsoft™) to determine *P* values by the unpaired student’s t-test.

## Results

### Distinct Pairwise Combinations of T Cell Independent (TI) and T Cell Dependent (TD) TD Signals for B Cell Activation and Class Switching

**Figure 1:**
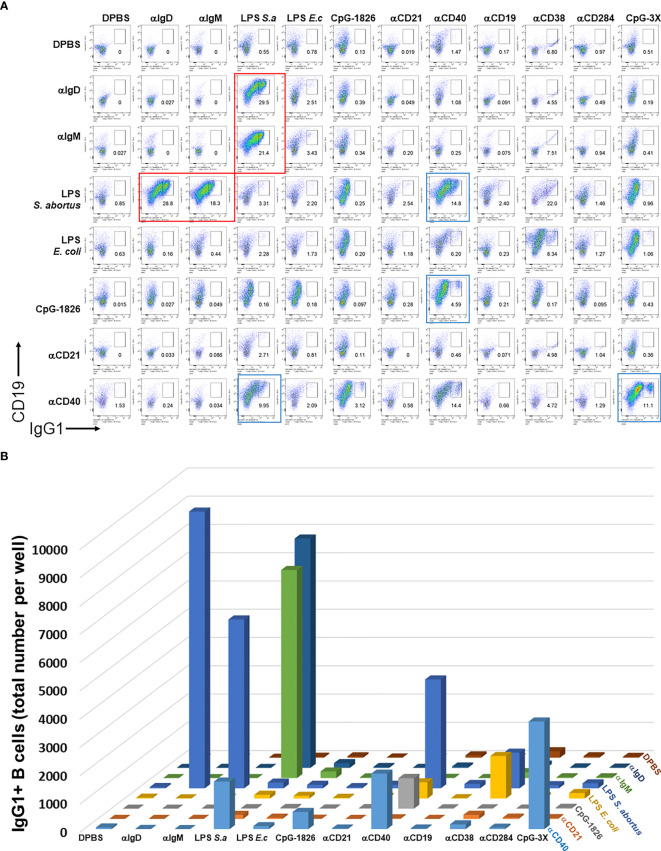
Distinct pairwise combinations of T-I and T-D signals for B cell activation and class switching. **(A)** Biotinylated T-I stimuli (0.15 μg/ml biotin-anti-IgD clone 11-26c, 0.15 μg/ml biotin-anti-IgM clone RMM1, 0.5 μg/ml biotin-LPS from either *E. coli* or *S. abortus equi*, 1.5 μg/ml biotin-CpG-1826, 5 μg/ml biotin-CpG-1826-3X, 0.5 μg/ml biotin-anti-CD19, 0.5 μg/ml biotin-anti-CD21, 0.5 μg/ml biotin-anti-CD284) and T-D stimuli (0.5 μg/ml biotin-anti-CD40, 0.5 μg/ml biotin-anti-CD38) were first mixed with 1 μg/ml streptavidin in each well of a V-bottom 96 well plate at rt for 2 hrs. DPBS was used to bring volume for each well of these biotinylated stimuli conjugated to streptavidin to a final 20 μl. They were then used to stimulate single cell suspensions (2.5 x 10^5^ cells/ml in each well) from mouse spleens in FBS-RPMI medium supplemented with 50 μM beta-mercaptoethanol (BME) and 25 ng/ml IL-4 in flat bottom 96 well plates for 4 days. Notice that the concentrations of stimuli can be converted to absolute amounts added per 250 μl V-well during the first biotin conjugation step by dividing by 4, e.g. 1 μg/ml streptavidin per cell culture means that 0.25 μg streptavidin was added to each well. Cells were then surface stained with antibodies to CD19, IgG1, and other B cell markers (not shown in these plots), and the viability dye 7AAD. Single viable B cells were plotted and the percent of IgG1^+^ cells is shown in each dark gate. Larger red rectangles show selected pairwise combinations that induce the most cell proliferation and CSR, which notably include biotinylated LPS + either anti-IgM, anti-IgD or CD40, and CpG + anti-CD40. **(B)** 3D chart showing the total number of IgG1^+^ B cells induced by pairwise combinations of stimuli from **(A)** above. Note that the first eight components in columns and rows are identical and therefore the assays for this 8 x 8 matrix is done in duplicate. This is particular prominent for the synergistic combination of LPS (*S. abortus equi*) and either anti-IgD or anti-IgM in the upper left corner; these correspond to the four plots delineated with red rectangles in **(A)**. Color coding is the same for all rows and varies for all columns.

We first set out to test the hypothesis that BCR and particular TI or TD stimuli synergize to induce B cell growth and antibody class switching. A large pairwise screen of common TI and TD stimuli showed that only a relatively small subset of them induced robust proliferation and CSR, which notably include biotinylated LPS + either anti-IgM, anti-IgD or CD40, and CpG + anti-CD40. Although previous reports first add biotinylated stimuli to B cell cultures, and then crosslink them with streptavidin, we chose to pre-mix the biotinylated stimuli first and then add them to B cell cultures. Biotinylated BCR-engaging reagents (anti-IgD, anti-IgM) T-independent stimuli (LPS from either *E. coli* or *S. abortus equi*, CpG-1826, CpG-1826-3X, anti-CD19, anti-CD21, anti-CD38, anti-CD284) and T-dependent stimulus (anti-CD40) were first mixed with 1 μg/ml streptavidin in each well of a V-bottom 96 well polypropylene plate at rt for 2 hrs. They were then used to stimulate single cell suspensions from mouse spleens in the presence of IL-4 in 96 well plates for 4 days. Cells were then surface stained with antibodies to CD19, IgG1, and other B cell markers (not shown in these plots). Single viable B cells were plotted and the percent of IgG1^+^ cells is shown in each dark gate. Red rectangles highlight selected pairwise combinations that induce the most cell proliferation and CSR, namely biotinylated LPS + either anti-IgM or anti-IgD, that are studied in more detail subsequently; blue rectangles identify plots which also show synergy of anti-CD40 and either LPS or CpG, but which are not followed up in this paper (**Figure 1A**). The quantification of the total number of IgG1^+^ B cells was also rendered as a 3D chart (**Figure 1B**). Replacing crosslinking of these biotinylated stimuli with streptavidin coated nanoparticles instead of just streptavidin also yielded similar results (**Supplementary Figure 1**). Since the LPS and IgM synergy stood out from the rest of the pairwise combinations, this suggested that for the remainder of this study we focus on further clarifying factors behind this synergy and whether particular parameters of crosslinking by streptavidin reagents are important.

### Streptavidin: Biotin Assembled Immunoconjugates Are Most Active Over A Narrow Concentration for Inducing CSR

**Figure 2:**
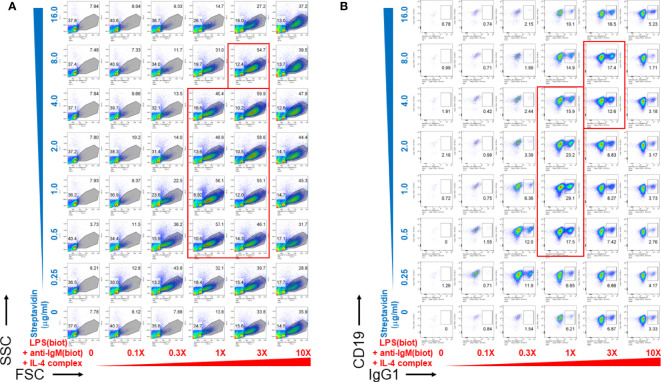
Pre-assembly of immunoconjugates is critical for their narrow dose-dependent B cell proliferation that correlates with class switching. Biotinylated LPS (S. abortus equi), biotinylated anti-IgM F(ab’)_2_, and anti-IL-4 mAb complexed with IL-4 were titrated along the columns (x-axis) of a 96 well V-bottom plate, whereas streptavidin was titrated along its rows (y-axis) in the indicated concentrations. Since LPS, anti-IgM and IL-4 were used at different concentrations for each well, the 1X (1-fold) concentration was: 1 μg/ml biotin-LPS, 0.2 μg/ml biotin-anti-IgM F(ab’)_2_, 10 ng/ml IL-4 complexed to 100 ng/ml biotin-anti-IL-4. The X-fold titration refers to multiples of this 1X cocktail of B cell stimulating stimuli. After pre-assembly of the biotinylated stimuli with streptavidin for 2 hrs, then were then added to the corresponding wells of a 96 well flat bottom plate containing splenocytes (2.5 x 10^5^ cells/ml in each well) in RPMI-FBS medium supplemented with BME. Cells were harvested after 4 days and stained with a cocktail of antibodies, including CD19 and IgG1. **(A)** Proliferation was visible as the increase in cell size in the forward scatter (FSC) channel, peaking at only certain ratios of biotinylated stimuli vs streptavidin (the most robust wells are included in the red rectangles). **(B)** CSR to IgG1 also peaked at only certain ratios of biotinylated stimuli vs streptavidin, which generally correlates with the extent of proliferation as in **(A)** (wells with the highest CSR are included in the red rectangles).

We then wanted to determine whether biotinylated LPS and anti-IgM were most effective as B cell growth and differentiation stimuli at particular concentration ranges of streptavidin crosslinking reagents. To do this, biotinylated LPS (from *S. abortus equi*), anti-IgM, and anti-IL-4 complexed with IL-4 were titrated along the columns (x-axis) of a 96 well V-bottom polypropylene plate, whereas streptavidin was titrated along its rows (y-axis) in the indicated concentrations (**Figure 2**). CSR to IgG1 also peaked at only certain ratios of biotinylated stimuli vs streptavidin (**Figure 2B**), which generally correlates with the extent of proliferation (**Figure 2A**). Since LPS, anti-IgM and IL-4 were used at different concentrations for each well, the 1X (1-fold) concentration was defined as: 1 μg/ml biotin-LPS, 0.2 μg/ml biotin-anti-IgM F(ab’)_2_, 10 ng/ml IL-4 complexed to 100 ng/ml biotin-anti-IL-4 mAb. The X-fold titration refers to multiples of this 1X cocktail of B cell stimulating stimuli. After our typical pre-assembly of the biotinylated stimuli with streptavidin for 2 hrs, they were then added to the corresponding wells of a 96 well flat bottom plate containing splenocytes in culture medium. Proliferation was visible as the increase in cell size in the forward scatter (FSC) channel, peaking at only certain ratios of biotinylated stimuli vs streptavidin (the most robust wells are included in the red rectangles) (**Figure 2A**). Likewise, CSR to IgG1 also peaked at only certain ratios of biotinylated stimuli vs streptavidin, generally correlates with the extent of proliferation as in **A** (wells with the highest CSR are included in the red rectangles) (**Figure 2B**). Interestingly, the maximal B cell growth and CSR occurred over a rather narrow range of streptavidin:biotin ratio at intermediate overall concentrations of stimuli. Even the highest concentration of unlinked stimuli (i.e. without streptavidin) could not compare in efficacy with the optimal streptavidn:biotin bound stimuli. Additional markers for germinal center B cells (PNA) and plasma cells (CD138) also had distinct optimal areas of concentration of the biotinylated stimuli vs streptavidin crosslinking, suggesting that signal strength differentially influences B cell fate along either the germinal center or plasma cell pathways (**Supplementary Figure 2**).

### Soluble Streptavidin or Streptavidin Nanoparticles Loaded With Biotinylated B Cell Stimuli Yield Dose-Dependent CSR

**Figure 3:**
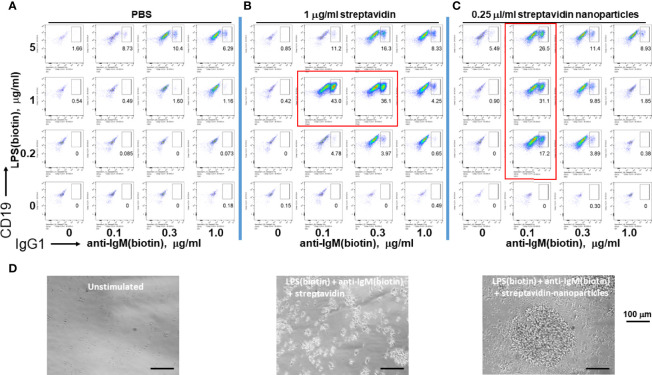
Assembly of immunoconjugates by either soluble streptavidin or streptavidin nanoparticles yields dose-dependent CSR. Since microorganisms like viruses and bacteria are nanometer to micron sized, soluble streptavidin or polystyrene nanoparticles (~ 100 nm in diameter) conjugated with streptavidin on their surface were used to conjugate biotinylated LPS and anti-IgM. **(A)** Biotin-anti-IgM was titrated in indicated amounts along the x-axis vs biotin-LPS along the y-axis in the wells of a V-bottom 96 well polypropylene plate for 2 hrs at rt. Each well was then added to the corresponding well of resting murine splenocytes (2.5 x 10^5^ cells/ml in each well) in RPMI-FBS plus BME containing 25 ng/ml IL-4. Cells were stained following our typical flow cytometry protocol (Methods) and the fluorescence of IgG1 vs CD19 were plotted. **(B)** Biotinylated anti-IgM F(ab’)_2_ and biotinylated LPS were first mixed together in individual wells of a V-bottom polypropylene plate, and then 0.25 μg/well (equivalent to 1 μg/ml in a 250 μl RPMI-FBS cell culture) streptavidin in DPBS was added in a final volume of 20 μl. **(C)** Streptavidin-conjugated polystyrene nanoparticles (~ 100 nm in diameter, Bangs labs) at 0.06 μl/well were added to premixed biotin-anti-IgM + biotin-LPS prior to each well being added to splenocyte cultures for 4 days prior to flow cytometry as before. (Preliminary studies tested these nanoparticles over a range of concentrations and found that they are not toxic to cells up to 0.25 μl/well). It is notable that both soluble streptavidin and streptavidin nanoparticles greatly enhance CSR to IgG1, but do so only at certain narrow combinations (plots within red rectangles) of biotinylated stimuli to streptavidin/streptavidin nanoparticles. **(D)** Phase contrast microscope images (20X) of unstimulated cells (left panel), splenocytes stimulated with conjugates of LPS(biotin), anti-IgM(biotin) and streptavidin coated nanoparticles (middle panel), or LPS(biotin), anti-IgM(biotin) and soluble streptavidin (right panel). Scale bar length is 100 micron.

Since microorganisms like viruses and bacteria are nanometer to micron sized, we tested whether soluble streptavidin or polystyrene nanoparticles (~ 100 nm in diameter) conjugated with streptavidin on their surface can effectively immobilize biotinylated LPS and anti-IgM to stimulate B cells. Both soluble streptavidin and streptavidin nanoparticles greatly enhanced CSR to IgG1, but only at certain narrow combinations of biotinylated stimuli to streptavidin/streptavidin nanoparticles. (**Figure 3**, plots surrounded by red rectangles). These results agree with the previous results using streptavidin variants to crosslink biotinylated stimuli. In this case, it is likely that the nanoparticles remain unaggregated and maintain their ~ 100 nm diameter. The batch of LPS used had a biotinylation ratio of 0.5 (i.e. half of the LPS molecules are biotinylated), and so would not be able to bind to more than one streptavidin nanoparticle. Anti-IgM F(ab)’2 is reported to typically have 2-3 biotins per molecule and so in principle can bind to more than one nanoparticle. However, this antibody fragment was used at ratios well below the binding capacity of the streptavidin nanoparticles and so it is unlikely that it is crosslinking two or more nanoparticles. Thus, it is possible that nanoparticles with diameter of about 100 nm loaded with tuned B cell stimuli are efficient reagents to induce B cell growth and class switching. One difference between using streptavidin vs streptavidin-coated nanoparticles is that the latter yields cell aggregates of larger size (i.e. having more cells per clump), though there are fewer numbers of such individual clumps, so that the overall cell expansion per well is comparable. We conclude that tuned aggregates of soluble streptavidin or solid nanoparticles conjugated with streptavidin can be used to bind biotinylated stimuli for enhanced B cell activation, and as such may be suitable platform in future immunization studies.

### Multimericities of Both BCR Engaging Anti-IgM and TLR4-Engaging LPS Are Critical for Enhanced B Cell Proliferation and CSR

**Figure 4:**
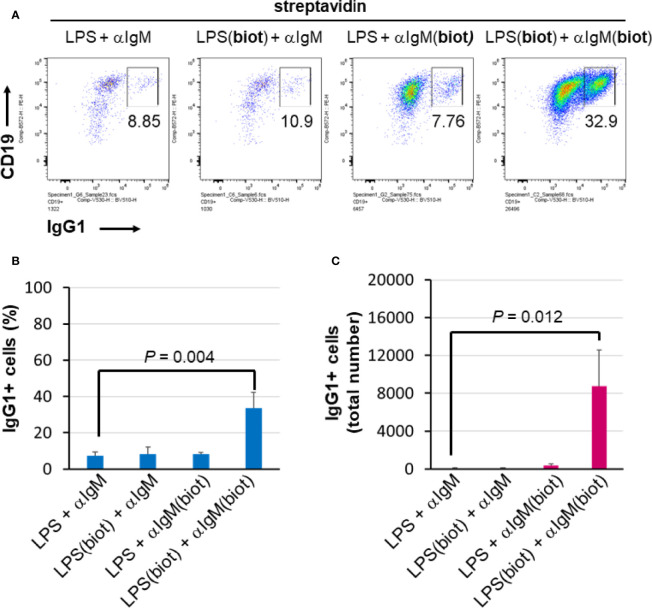
Multimericities of BCR engaging anti-IgM and TLR4-engaging LPS are critical for enhanced B cell proliferation and CSR. **(A)** To distinguish whether multimerization of LPS or anti-IgM is responsible for the enhanced effect at inducing B cell proliferation and CSR, unbiotinylated or biotinylated LPS (0.3 μg/ml = 0.075 μg/ml per well), and anti-IgM F(ab’)_2_ (0.1 μg/ml = 0.025 μg/ml per well) were conjugated to streptavidin (1 μg/ml = 0.25 μg/ml per well) in some wells of a 96-well V-bottom polypropylene plate. These streptavidin:biotin immunoconjugates were then added to splenocyte cultures (2.5 x 10^5^ cells/ml in each well) in 96 well flat bottom tissue culture plates in RPMI-FBS/BME, also in the presence of 50 ng/ml IL-4, for 4 days. Flow cytometry of representative results indicates that biotinylation of both LPS and anti-IgM is required for maximal CSR to IgG1 in this system. **(B)** Quantification of cell proliferation in the four conditions described above. **(C)** Quantification of CSR to IgG1 in the four conditions described above. It is notable that maximal proliferation and CSR occurs only when both LPS and IgM are biotinylated and premixed to streptavidin.

To distinguish whether multimerization of LPS or anti-IgM is responsible for the enhanced effect at inducing B cell proliferation and CSR, unbiotinylated or biotinylated LPS and anti-IgM were conjugated to streptavidin in polypropylene V-bottom plates as before prior to being added to splenocyte cultures in flat 96 well plates. The results show that maximal proliferation and CSR occurs only when both LPS and anti-IgM are biotinylated and premixed to streptavidin (**Figure 4A**). Both the relative class switching to IgG1 (**Figure 4B**) and the overall number of IgG1^+^ B cells (**Figure 4C**) were largest when both LPS and anti-IgM were biotinylated. Note that the relatively low concentrations of LPS and IgM chosen meant that overall B cell growth is low (**Figure 4A**, three left plots) unless these reagents are multimerized with streptavidin (**Figure 4A**, rightmost plot). With our present methods it is not possible to evaluate the composition of streptavidin complexes, though it is likely that an ensemble of single streptavidins as well as biotin:streptavidin aggregates exists containing combinations of 0, 1, 2, 3 of 4 molecules of LPS and anti-IgM as the active agents.

### IL-5 Enhances the Viability of B Cell Cultures Stimulated With LPS, Anti-IgM and IL-4

We then tested whether LPS and anti-IgM conjugates enhance B cell growth and class switching even under cell culture viability conditions improved by other means. Reports in the literature describe a pro-survival role for IL-5 in *in vitro* B cell cultures (31, 32). Biotinylated LPS, anti-IgM F(ab’)2, and anti-IL-4 complexed with IL-4 were titrated along the columns (x-axis) of a 96 well V-bottom polypropylene plate (**Supplementary Figure 3**). Cell blasting and viability were considerably enhanced by IL-5. At low concentrations of LPS + anti-IgM + IL-4, IL-5 promoted plasma cell/plasmablast differentiation, consistent with well-known studies (47). However, this effect is largely abrogated by high concentrations (10X) of these stimuli, likely due to a high level of BCR signaling, which is reported to attenuate or delay plasma cell differentiation (48, 49) until the conclusion of the germinal center B cell differentiation pathway. Indeed, the high (10X) concentration of above stimuli drive excellent B cell proliferation, viability and CSR. Thus, IL-5 was used in some subsequent studies to improve the viability of the *in vitro* cell cultures.

### Streptavidin, PEG(Streptavidin)8 or Traptavidin Enhance B Cell Growth and CSR

**Figure 5:**
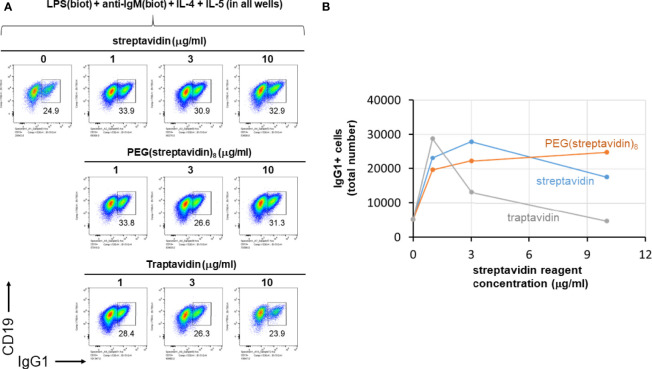
Streptavidin, PEG(streptavidin)8 or traptavidin enhance B cell growth and CSR. LPS(biotin) (2 μg/ml) and anti-IgM F(ab’)_2_ (0.3 μg/ml) biotin-LPS were incubated with the indicated amounts of streptavidin, PEG(streptavidin)8, or traptavidin in the wells of a V-bottom polypropylene plate for 2 hrs at rt (all wells also contained 50 ng/ml IL-4 and 50 ng/ml IL-5). Each well was then added to the corresponding well of resting murine splenocytes (1.5 x 10^5^ cells/ml in each well) in RPMI-FBS plus BME containing. After 4 days in culture, cells were analyzed by flow cytometry. **(A)** The growth of B cells visualized by the typical forward vs side scattering indicates better growth at intermediate streptavidin:biotin conjugate ratios. **(B)** Quantification of the total number of IgG1+ B cells per well for each of the avidin reagent concentrations shown in **(A)** above.

We next further tested the hypothesis that streptavidin is acting mainly as a crosslinker of biotinylated stimuli and not *via* other mechanisms affecting B cells. To do this, LPS(biotin) and anti-IgM F(ab’)2 were incubated with the indicated amounts of streptavidin, PEG(streptavidin)8, or traptavidin pre-assembled in the wells of a V-bottom plate (all wells also contained IL-4 and IL-5). The growth of B cells visualized by the typical forward vs side scattering indicates better growth at intermediate streptavidin:biotin conjugate ratios (**Supplementary Figure 4**). The increased B cells growth associated with intermediate streptavidin:biotin conjugates correlates with increased CSR to IgG1 (**Figure 5**). B cell growth and CSR were lower at the ends of the titration, i.e. without or with the maximum amount of the particular streptavidin variant. While cell growth and CSR in the streptavidin and traptavidin titrations peak at intermediate (1-3 μg/ml) concentrations of these biotin-crosslinking reagents, PEG(streptavidin)8 plateaus at 3-10 μg/ml, possibly since each streptavidin-PEG octamer does not conjugate to other octamers to form larger PEG-streptavidin:biotin multimers. Other experiments with neutravidin (a deglycosylated version of egg avidin) showed the same pattern of enhanced B cell growth and class switching at particular ratios of neutravidin to biotinylated stimuli (not shown). Thus, the enhancement of B cell growth and CSR does not depend uniquely on streptavidin (which is used throughout this paper), but rather on the biotin binding, and therefore, crosslinking ability of the streptavidin/avidin family. In other control experiments, titrating in soluble D-biotin to concentrations of streptavidin prior to adding in LPS(biotin) and anti-IgM(biotin) abrogated the enhancing effect of streptavidin on B cell proliferation and CSR to IgG1. Taken together with the data comparing unlabeled vs biotinylated LPS and anti-IgM (**Figure 4**), it is most plausible that forming streptavidin:biotin conjugates and aggregates exerts dose-dependent effects on the strength of biotinylated B cell stimuli for enhanced B cell growth and differentiation.

### Streptavidin-Biotin Immunoconjugates Must Be Over 300 kDa According to Molecular Sieve Filtration Followed by *In Vitro* Cell Stimulation

**Figure 6:**
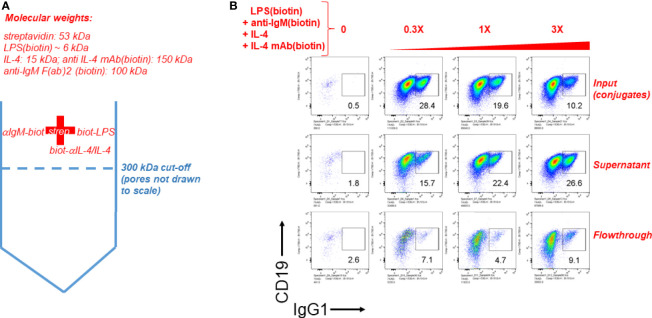
Streptavdin-biotin immunoconjugates must be over 300 kDa according to molecular sieve filtration followed by cell stimulation. **(A)** Schematic of the experiment to filter streptavidin:biotin B cell immunoconjugates through a molecular sieving centrifugal filter unit with 300 kDa MW cut-off membrane pores. Since IL-4 is essential for B cell growth and CSR to IgG1 it was tethered by its biotinylated antibody at a 1:1 ratio. **(B)** Biotinylated LPS (3 μg/ml), biotinylated anti-IgM F(ab’)_2_ (0.5 μg/ml) and biotin-anti-IL-4 0.5 μg/ml in complex with IL-4 (50 ng/ml) were conjugated to streptavidin (3 μg/ml). This mixture is referred to as input. The input was centrifuged through a Vivaspin 6 ultrafiltration (molecular sieving) filter with a 300 kDa cut-off, and the supernatant and flow-though were collected. The supernatant volume was brought to be equal to the volume of flow-through by adding DPBS. Next, equal volumes of input, supernatant and flow-through were added to splenocyte cultures at 0, 0.3X, 1X, and 3X, where 1X refers to the initial concentration of each component in the input. CSR to IgG1 after 4 days of stimulation reveals that most of the CSR-inducing activity is retained in the supernatant, but is lost in the flow-through.

Finally we tested the notion that streptavidin aggregates (or streptavidin bridges) formed by soluble streptavidin tetramer conjugated to biotinylated LPS (typical molecular weight range is 50-100 kDa) and anti-IgM F(ab’)_2_ (molecular weight is ~ 100 kDa) consist of complexes of more than one of these individual stimuli. To do this, biotinylated LPS, biotinylated anti-IgM F(ab’)_2_ and biotinylated anti-IL-4 mAb in complex with IL-4 were conjugated to streptavidin, and this mixture is referred to as input. Since IL-4 is essential for B cell growth and CSR to IgG1 it was tethered by its biotinylated antibody at a 1:1 ratio, which was determined in our other studies (not shown) not to interfere with IL-4 activity, but rather act as a carrier similar to the original report on IL-4 antibody complexes (50) and the subsequently reported tethering of IL-2 by its antibody at 1:1 stoichiometry (51). The input was centrifuged through a Vivaspin 6 ultrafiltration (molecular sieving) filter with a 300 kDa cut-off, and the supernatant and flow-though were collected. The supernatant volume was brought to be equal to the volume of flow-through by adding DPBS. A schematic of the procedure is shown in **Figure 6A**. Next, equal volumes of input, supernatant and flow-through were added to splenocyte cultures at 0, 0.3X, 1X, and 3X, where 1X refers to the initial concentration of each component in the input. CSR to IgG1 after 4 days of stimulation reveals that most of the CSR-inducing activity is retained in the supernatant, but is lost in the flow-through, leading to the conclusion that the immunoconjugates must be more than ~ 300 kDa in size (**Figure 6B**). Since the biggest individual components are biotin-anti-IgM and biotin-anti-IL-4 (~150 kDa), and since IL-4 is required for B cell proliferation, the results strongly suggest that all components are attached to streptavidin to make conjugates > 300 kDa. From this we conclude that it is likely that this is a collection of aggregates of different composition of overall MW, most of which are well above the MW of a single antibody fragment or LPS molecule. Further studies are under way to characterize the molecular weight distribution of these streptavidin:biotin aggregates.

## Discussion

The families of receptors that need to be actively combined to yield desired responses have so far been partially determined for the main immune cell types, and especially for T cell (52) and B cell subsets (1, 53, 54). B cell activation requires, at a minimum, either one of three receptor types: BCR, PRRs or CD40. The ensuing outcomes, i.e. extent of cell enlargement, proliferation, the antibody isotype expressed, other differentiation markers along the germinal center, memory or plasma cell pathway, depend on the strength and duration of the various activated receptors (37–40). Their combinations can synergize (39, 55–57), and IL-4 acts as a growth factor to promote cell differentiation (and additionally enhances or induces immunoglobulin class switch recombination to IgG1 and IgE isotypes). In our present study, we first did an unbiased screen of BCR, PRR and cytokine receptors to measure any potential synergy in inducing B cell growth and antibody class switching. LPS and anti-IgM/IgD stood out as the most synergistic combination of stimuli, followed by anti-CD40 plus CpG. Although in this report we only analyzed the use of IL-4 as a general B cell growth factor and promoter of CSR to IgG1, the potentiating effect that multimeric presentation of ligands for TLR4 and BCR has on B cell activation and CSR is expected to hold for other cytokines (1) that induce or promote class switching to the other immunoglobulin isotypes.

The main aim of the present study was influenced by the body of literature proving or suggesting that the engagement of multiple copies (17) of immune receptors in relatively stable patches of nanoscale domains (8) (signalosomes), and the combination of different types of receptor families (1, 43, 58, 59) are required for full immune cell mobilization, i.e. activation, proliferation and differentiation. An early report stimulated B cells *in vitro* with biotinylated anti-IgM or anti-IgD followed 30 min later by hypercrosslinking them with avidin. This report also showed that IL-4 or anti-CD40 could synergize with the strong BCR crosslinking biotin:avidin reagents to rescue B cells from apoptosis and induce their enlargement and DNA synthesis (30). In our previous publications, we also stimulated B cells with TLR agonist and anti-IgD conjugated to dextran strands, though we did not study how crosslinking of either a TLR agonist, an anti-IgM/IgD antibody or both compare to the soluble version (39, 56). In the present study, we wanted to find out whether a method to link both BCR and TLR4 agonists could be developed that is accessible and relatively straightforward and could facilitate culturing and differentiation of B cells *in vitro* as a means to eventually predict PAMP adjuvanticity when part of vaccines *in vivo*.

A recent report described the preparation of lipid vesicles containing embedded dsDNA that displayed biotin at the terminus, which could adhere to flat supported lipid bilayer membranes (also containing embedded biotinylated dsDNA) upon bridging streptavidin; effective adherence was observed with streptavidin concentrations ranging widely up to about three orders of magnitude (60). In comparison with this report, our biotinylated stimuli were maximally active in their induction of B cell growth and CSR to IgG1 over a rather narrow range of streptavidin crosslinking for any given concentration of biotinylated stimuli. In another report, B cells were stimulated with biotinylated anti-IgM and then hypercrosslinked with streptavidin to trigger BCR signaling as reported by cell size increases and DNA synthesis after three days (30). While inspiring the present work, this previous report used much higher amounts of biotinylated anti-IgM and (strept)avidin (30) without measuring B cell class switching. We think that the narrow range of streptavidin to biotin ratio observed to be optimal for B cell growth and CSR is due to the fact that multimers of LPS and anti-IgM conjugates may arise only at narrow biotin: streptavidin crosslinking ratios. Streptavidin reagents used in mostly diagnostic assays are typically added in excess, and depending on conditions, may give rise to multimeric aggregates referred to as streptavidin bridges (61). The narrow streptavidin: biotin ratio in our system is reminiscent of the antibody: antigen precipitin reaction (62) which also peaks at relatively a narrow range (zone of equivalence) of antibody to antigen to give rise to a large scale lattices of repetitive antibody: antigen pairs.

Our B cell stimulation assays *in vitro* revealed that crosslinking of biotinylated LPS and anti-IgM by streptavidin, or immobilized on nanoparticle coated with streptavidin, needs to be empirically ‘tuned’ for it to result in higher B cell growth and differentiation as measured by antibody class switching. Although we cannot disentangle the distinct mechanisms by which tuned immunoconjugates robustly activate B cells, among the main ones are crosslinking the BCR *in cis* (in one cell) and crosslinking the BCR *in trans* (bridging two or more cells). The first mechanism is the one usually discussed in the literature, as highlighted in our references in the Introduction, and briefly below.

There are multiple reasons why multimeric antigens, such as those on the surface of viruses, bacteria or synthetic nanoparticles and immunoconjugates are reported to trigger sustained BCR signaling, whereas soluble, monovalent antigens are not thought to engage the BCR as well. As a simple approximation, engaging multiple BCRs by arrayed antigen is reminiscent of linear free energy relationships in protein-ligand pairs. According to this view, free energies of multiple receptor: ligand pairs can be considered to be additive up to an extent and depending on the system, particularly if the units are flexible and independent (63) rather than rigid or sterically interfering with each other (64). Typical multimeric ligands include IgG and IgE antibodies with two identical binding arms, pentameric/hexameric IgM antibodies, dimeric IgA antibodies, antibodies; lectins, and the numerous viruses and bacteria with their repetitive ligands binding to the corresponding cell targets. Cell-cell interactions themselves involve many copies of one or more types of receptor pairs. In general, cooperative binding of different ligands to different, but adjacent, receptors is widespread in biology, and is exemplified by cooperative binding of various transcription factors and RNA polymerase (65), and cooperative binding of epigenetic factors to particular chromatin modules (66). Another familiar analogy is the melting temperature (a reflection of the degree of strand complexation) of nucleic acid duplexes, which for typical buffers and temperatures has a clear relationship based on the GC content and the total number of base pairs up to about 80 nt, after which it remains steady (67, 68). In all these systems, adding multiple, relatively independent and flexible units increases the overall stability of ligand receptor pairs (provided multiple ligands do not sterically interfere with one another), resulting in enhanced multimeric affinity, i.e. avidity (69, 70) or attinebility (71), compared to a collection of single ligands distant from one another.

Cell surface receptors present additional complexities compared to isolated biochemical systems. The cell membrane, while idealized as a 2D surface, can have ruffles, is influenced by the underlying cytoskeleton, and can undergo fluxes of removal and creation by endocytosis and receptor synthesis, respectively. Furthermore, strong binding resulting in a long residence of ligand on receptor (72) (i.e. stable or long lifetime of complex) is necessary but not sufficient to trigger the pathways downstream of the receptor. In the words of Bindslev (73): “just because there is a conformational change when a ligand binds, this conformational change is not necessarily the one which activates the receptive unit for function.” A key example for immune cells would be whether or not the receptor is mechanosensitive (74), i.e. requires some type of mechanical force or even vibration by the ligand to trigger the receptor. The BCR is known to be a mechanosensitive receptor (75), whereas it is unclear whether TLR4 and cytokine receptors are also mechanosensitive. Engaging the BCR by either naturally occurring multimeric antigen on pathogen surfaces, or by synthetic approaches using nanoparticles and immunoconjugates (as in the present study) is expected to not only form a larger BCR signalosome but possibly also provide the mechanoactive transduction it requires. Note that while traditionally it was thought that crosslinking, or aggregation of individual inactive BCR monomer units triggers BCR signaling, there is recent evidence that in fact antigen-mediated dispersion of pre-formed oligomeric BCR units into active monomers is the triggering mechanism (76); regardless of either scenario, higher multivalent antigens are expected to more efficiently trigger BCR signalosome formation and consequently BCR signaling compared to low valency or monovalent antigens.

A bystander benefit of using anti-IgM aggregates as in the present study is the possibility of crosslinking different B cells; indeed, B cells stimulated with LPS and anti-IgM biotin:streptavidin aggregated typically form clumps of cells, unlike B cells stimulated with soluble monomeric CpG (77). These could be thought of as arising from homotypic adhesion of cells mainly *via* integrin receptors, but they could also be induced by multimeric stimuli coating the membranes of two different cells, eventually producing the typically spheroids of hundreds of cells (78, 79) (**Figure 3D**). Thus, B cells naturally form adhesion clusters, and multimeric ligands used to stimulate them may facilitate multicellular cluster formation, i.e. coating of one cell with multivalent stimuli renders them more effective ligands for signaling *in trans* (to other cells). The situation *in vivo* is somewhat similar with follicles of B cell centroblasts extracting antigen in the dark zone before migrating as centrocytes to the light zone to get additional T cell help (80), as well as DC help and multimeric BCR signals by antigen bound by IgM which themselves are multimerized *via* binding FcμR on DC membranes and dendrites (4). Our experiments with conjugates of biotinylated stimuli and either streptavidin, 8-arm polyethylene glycol (PEG) dendrimer conjugated with 8 streptavidins, or the stabler binding streptavidin variant called traptavidin showed similar profiles of enhancement of B cell class switching. The LPS and anti-IgM immunoconjugates had a molecular weight higher than 300 kDa as measured by molecular sieving experiments. Since streptavidin:biotin bridges or aggregates are heterogeneous (61), it is not currently possible to delineate whether the activity requires crosslinking of at least two different cells (*in trans*) or whether it is inducing larger patches of BCR and/or TLR4 signalosomes in one cells (*in cis*). The typical B cell proliferation and CSR were comparable for both streptavidin immuniconjugates and streptavidin nanoparticle immunoconjugates, though the latter gave rise to fewer but larger cell clumps. Performing single cell experiments (i.e. one cell per well) in future studies should address the question of whether extent of engagement of multiple cells influences B cell responses.

The enhancement of B cell responses by multimericity is expected to apply to other TLR agonists, especially those that are naturally part of microbial cell walls and occur in multiple copies, and possibly to nucleic acid sensing PRRs. In this context, it was recently reported that activation of cGAS sensor component of the cGAS-STING pathway depends significantly on increasing the substrate DNA length (81). Whether multimericity enhances the signaling of non-PRRs, especially the important TNF family is unclear. These receptors are typically membrane homotrimers engaging the corresponding trimeric ligands, and a synthetic 60-mer form of BAFF has been reported to be more active than the soluble trimeric form (82). Since host membrane receptors may be *de facto* multimeric due to the 2D membrane positioning, it is probable that large assemblies on the order of nanodomains or macrodomains are more active than single receptors (23).

In conclusion, the present report focused on the key enhancement that multimeric ligand engagement of TLR4 and BCR receptors for non-endogenous ligands has on B cell activation and class switching. While we focused on anti-IgM as the reagent of choice to trigger BCR signaling, anti-IgD is expected to be at least equally efficient since IgD is expressed at higher levels than IgM, anti-IgD-dextran reagents (25, 27, 31, 32) are well known to trigger the BCR. In addition, several reports have highlighted the unique responsiveness of IgD to polyvalent antigens to more efficiently guide B cells along the germinal center pathway (83–85); our ongoing studies comparing multimeric anti-IgD, anti-IgM and anti-CD79α/β will be reported elsewhere.

In general, the role that multimeric ligand engagement has on all types of immune receptors, including those binding endogenous membrane or soluble ligands, remains an important topic of research due to the dual chemical (namely, free energy) and biological (namely cooperativity and emergent behavior) sources that underpin multimericity (70). So, even the experimentally ubiquitous streptavidin:biotin system can be versatile in making custom immunoconjugates that, while not precise in their geometry and molecular weight (61), are quite potent and selective activators of particular cell functions, provided all components are tuned for a synergistic whole. In general, research on the oligomeric form of both antigen and co-stimulatory PAMPs and other adjuvants are expected to remain wide avenues of experimental immunology, and particularly for vaccine research.

## Data Availability Statement

The raw data supporting the conclusions of this article will be made available by the authors, without undue reservation.

## Ethics Statement

The animal study was reviewed and approved by Institutional Animal Care and Use Committee University of California, Irvine, and by the Animal Care and Use Review Office (ACURO) of the U.S. Army Medical Research and Materiel Command (USAMRMC).

## Author Contributions

EP, PF, and DD designed the study, analysed the data, and wrote the manuscript. EP performed the experiments. JH-D, SJ, and ES discussed the data, analysed information in the literature, provided references and wrote the manuscript. All authors contributed to the article and approved the submitted version.

## Funding

This work was supported by Department of Defense (DoD) grants HDTRA1-16-C-0009 (PI: PF) and HDTRA11810035 (PI: PF), DoD grant HDTRA11810036 (PI: DHD), NIH grant 1U01AI160397 (PI: DHD).

## Conflict of Interest

All of the authors own shares in Nanommune Inc. Nanommune does not sell any products described in this paper, nor funded any part of the work described herein. Neither Nanommune nor its shareholders are likely to benefit from the results described in this publication.

## Publisher’s Note

All claims expressed in this article are solely those of the authors and do not necessarily represent those of their affiliated organizations, or those of the publisher, the editors and the reviewers. Any product that may be evaluated in this article, or claim that may be made by its manufacturer, is not guaranteed or endorsed by the publisher.
