# Supplementary material for: Multimericity Amplifies the Synergy of BCR and TLR4 for B Cell Activation and Antibody Class Switching
Source: Front Immunol. 2022 May 19;13:882502. doi: 10.3389/fimmu.2022.882502 (PMC9161726; doi:10.3389/fimmu.2022.882502)
Supplement: Supplementary file 1 [file DataSheet_1.docx]

Supplementary Material

## Supplementary Figures 1-4

**
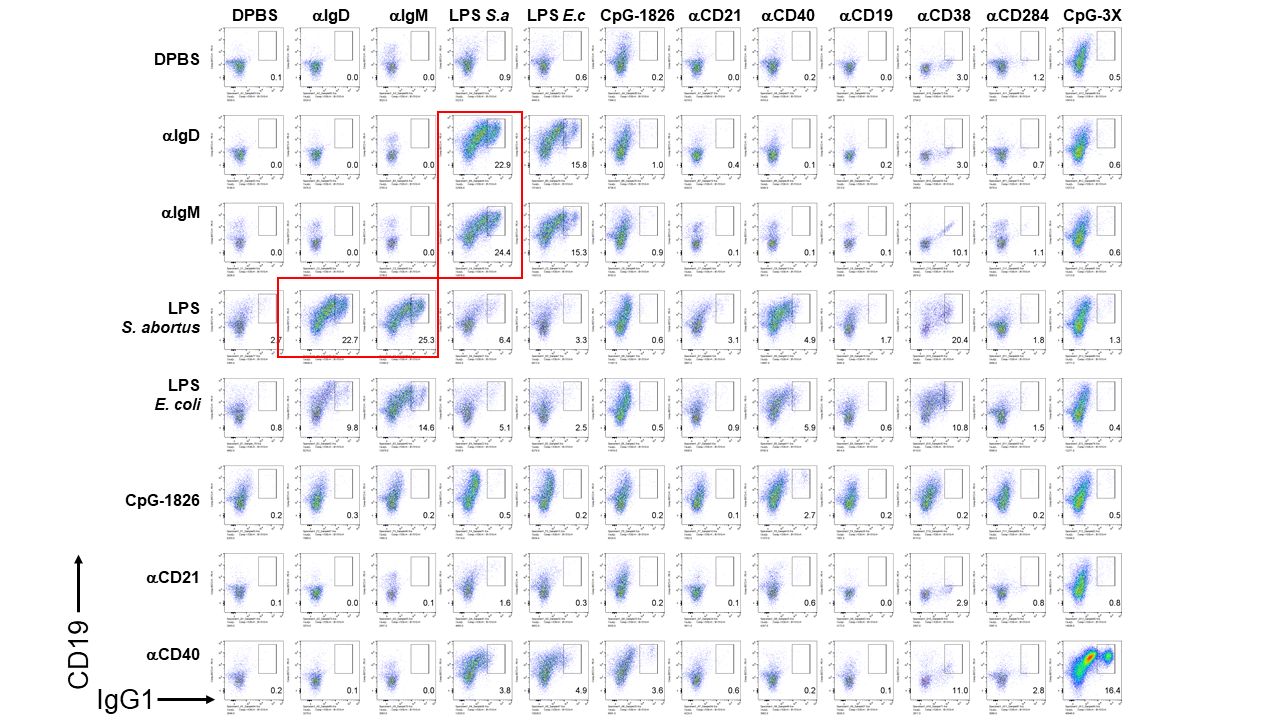
**

**Suppl. Figure 1. Distinct pairwise combinations of T-I and T-D signals on nanoparticles for B cell activation and class switching.** Biotinylated T-I stimuli (0.15 μg/ml biotin-anti-IgD clone 11-26c, 0.15 μg/ml biotin-anti-IgM clone RMM1, 0.5 μg/ml biotin-LPS from either E. coli or S. abortus equi, 1.5 μg/ml biotin-CpG-1826, 5 μg/ml biotin-CpG-1826-3X, 0.5 μg/ml biotin-anti-CD19, 0.5 μg/ml biotin-anti-CD21, 0.5 μg/ml biotin-anti-CD284) and T-D stimuli (0.5 μg/ml biotin-anti-CD40, 0.5 μg/ml biotin-anti-CD38) were first mixed with 0.2 μl/ml of streptavidin-coated polystyrene nanoparticles 103 nm in diameter (Bangs Laboratories, cat # CP01000) in each well of a V-bottom 96 well plate at rt for 2 hrs. Note that nanoparticles are provided as 1% solids, and 0.2 μl/ml refers to this 1% slurry of nanoparticles (equivalent to 0.002 μl/ml absolute concentration of solid nanoparticles). DPBS was used to bring volume for each well of these biotinylated stimuli conjugated to streptavidin to a final 20 μl. They were then used to stimulate single cell suspensions (2.5 x 10^5^ cells/ml in each well) from mouse spleens in FBS-RPMI medium supplemented with 50 μM beta-mercaptoethanol (BME) and 25 ng/ml IL-4 in flat bottom 96 well plates for 4 days. Notice that the concentrations of stimuli can be converted to absolute amounts added per 250 μl V-well during the first biotin conjugation step by dividing by 4, e.g. 1 μg/ml streptavidin per cell culture means that 0.25 μg streptavidin was added to each well. Cells were then surface stained with antibodies to CD19, IgG1, and other B cell markers (not shown in these plots), and the viability dye 7AAD. Single viable B cells were plotted and the percent of IgG1^+^ cells is shown in each dark gate. Larger red rectangles show selected pairwise combinations that induce the most cell proliferation and CSR, which notably include biotinylated LPS + either anti-IgM, anti-IgD or CD40, and CpG + anti-CD40.


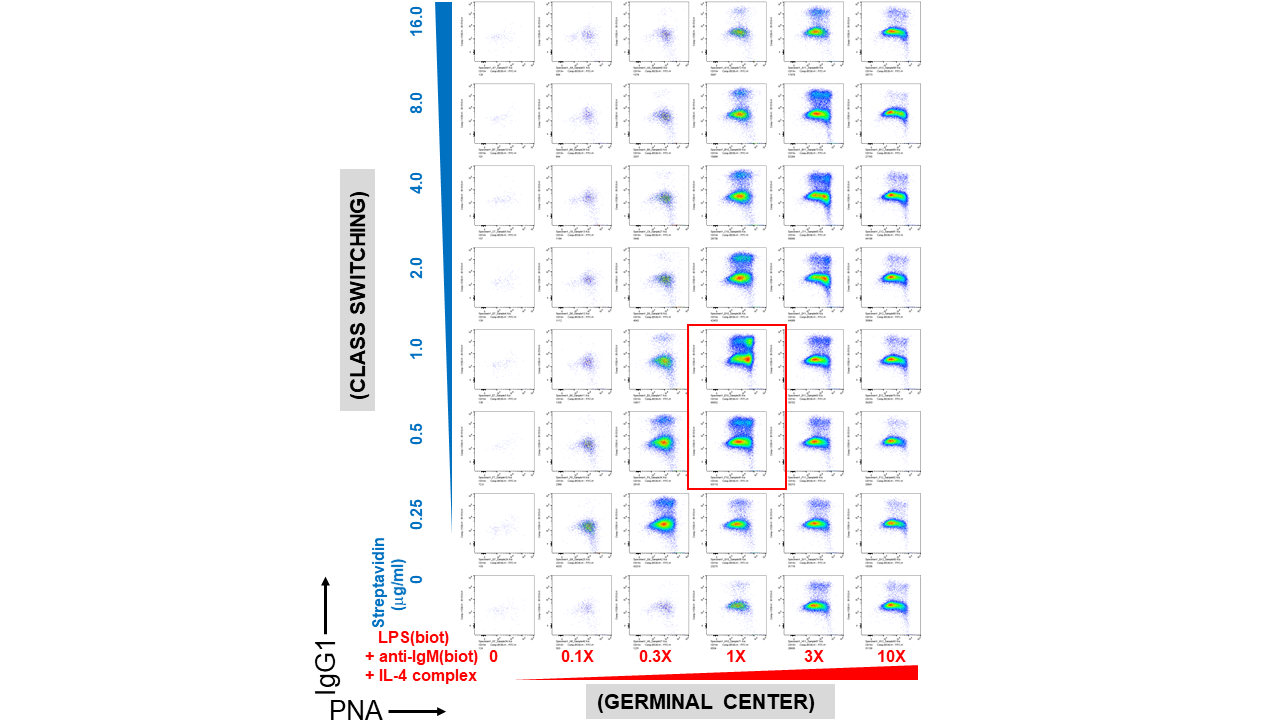


**Suppl. Figure 2 (A). Pre-assembly of immunoconjugates is critical for their narrow dose-dependent B cell proliferation that correlates with their expression of the germinal center biomarker PNA.** Biotinylated LPS (S. abortus equi), anti-IgM, and anti-IL-4 complexed with IL-4 were titrated along the columns (x-axis) of a 96 well V-bottom polypropylene plate, whereas streptavidin was titrated along its rows (y-axis) in the indicated concentrations. Since LPS, anti-IgM and IL-4 were used at different concentrations for each well, the 1X (1-fold) concentration was: 1 μg/ml biotin-LPS, 0.2 μg/ml biotin-anti-IgM F(ab’)_2_, 10 ng/ml IL-4 complexed to 100 ng/ml biotin-anti-IL-4. The X-fold titration refers to multiples of this 1X cocktail of B cell stimulating stimuli. After pre-assembly of the biotinylated stimuli with streptavidin for 2 hrs, then were then added to the corresponding wells of a 96 well flat bottom plate containing splenocytes (2.5 x 10^5^ cells/ml in each well) in RPMI-FBS medium supplemented with BME. Cells were harvested after 4 days and stained with a cocktail of antibodies, including CD19 and IgG1. (**A**) Germinal center character of the B cells was indicated by expression of PNA (x-axis), which correlated with class switching to IgG1 (y-axis), and peaked at only certain ratios of biotinylated stimuli vs streptavidin (the most robust wells are included in the red rectangles).

**
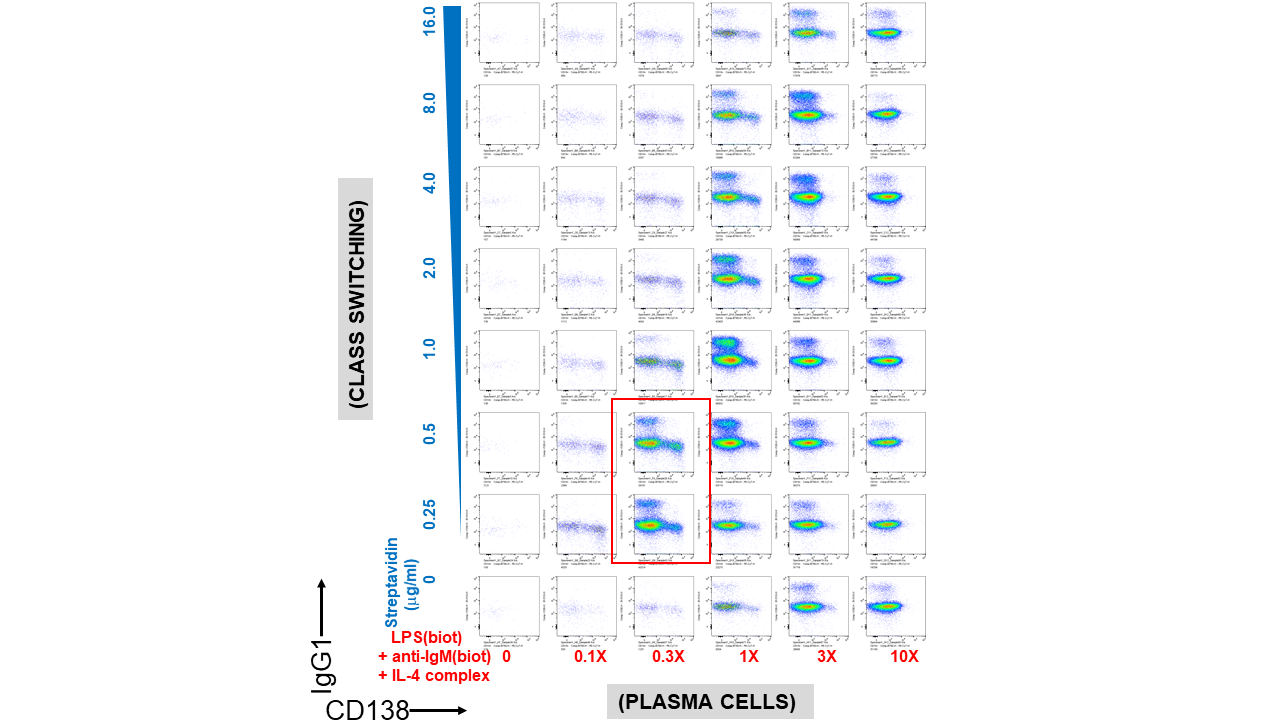
**

**Suppl. Figure 2 (B). Pre-assembly of immunoconjugates is critical for their narrow dose-dependent B cell proliferation that correlates with their expression of the plasma cell/plasmablast biomarker CD138.** Biotinylated LPS (S. abortus equi), anti-IgM, and anti-IL-4 complexed with IL-4 were titrated along the columns (x-axis) of a 96 well V-bottom polypropylene plate, whereas streptavidin was titrated along its rows (y-axis) in the indicated concentrations. Since LPS, anti-IgM and IL-4 were used at different concentrations for each well, the 1X (1-fold) concentration was: 1 μg/ml biotin-LPS, 0.2 μg/ml biotin-anti-IgM F(ab’)_2_, 10 ng/ml IL-4 complexed to 100 ng/ml biotin-anti-IL-4. The X-fold titration refers to multiples of this 1X cocktail of B cell stimulating stimuli. After pre-assembly of the biotinylated stimuli with streptavidin for 2 hrs, then were then added to the corresponding wells of a 96 well flat bottom plate containing splenocytes (2.5 x 10^5^ cells/ml in each well) in RPMI-FBS medium supplemented with BME. Cells were harvested after 4 days and stained with a cocktail of antibodies, including CD19 and IgG1. Plasma cell formation also peaked at only certain ratios of biotinylated stimuli vs streptavidin, which generally correlates with the extent of proliferation as in Fig. 2A of main text. Two representative wells with high proliferation are indicated by red rectangles.

**
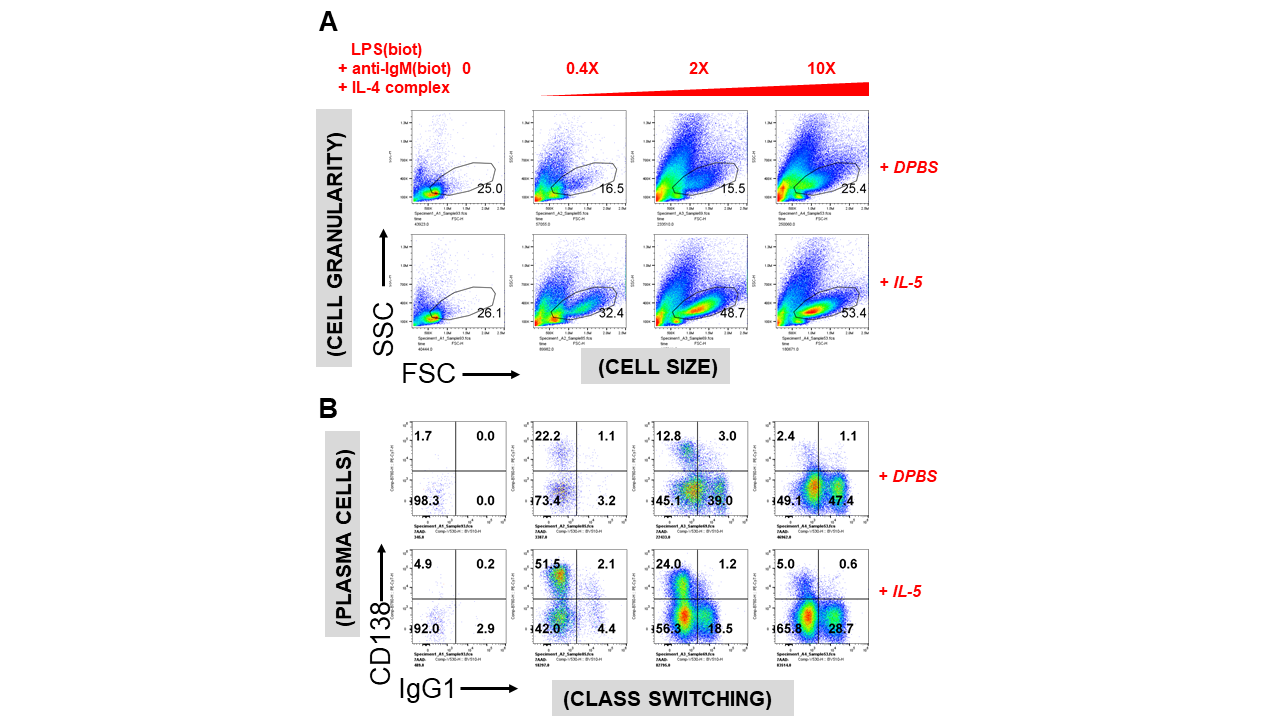
**

**Suppl. Figure 3. IL-5 enhances the viability of B cell cultures stimulated with LPS, anti-IgM and IL-4 and promotes IgM^+^ plasma cells.** Biotinylated LPS, anti-IgM F(ab’)_2_, and anti-IL-4 complexed with IL-4 were titrated along the columns (x-axis) of a 96 well V-bottom polypropylene plate at 0, 0.4X, 2X and 10X, where 1X (1-fold) concentration was defined as: 1 μg/ml biotin-LPS, 0.2 μg/ml biotin-anti-IgM F(ab’)_2_, 20 ng/ml IL-4 complexed to 200 ng/ml biotin-anti-IL-4. **(A)** Cell blasting and viability were considerably enhanced by IL-5. **(B)** At low concentrations of inducing stimuli (LPS + anti-IgM + IL-4), IL-5 promoted plasma cell/plasmablast differentiation (CD138 expression along the y-axis in each plot), consistent with well-known studies. However, this plasma cell promoting effect is largely abrogated by high concentrations (10X) of these stimuli, while the excellent cell proliferation, viability and CSR to IgG1 (expression along the x-axis in each plot) is retained.


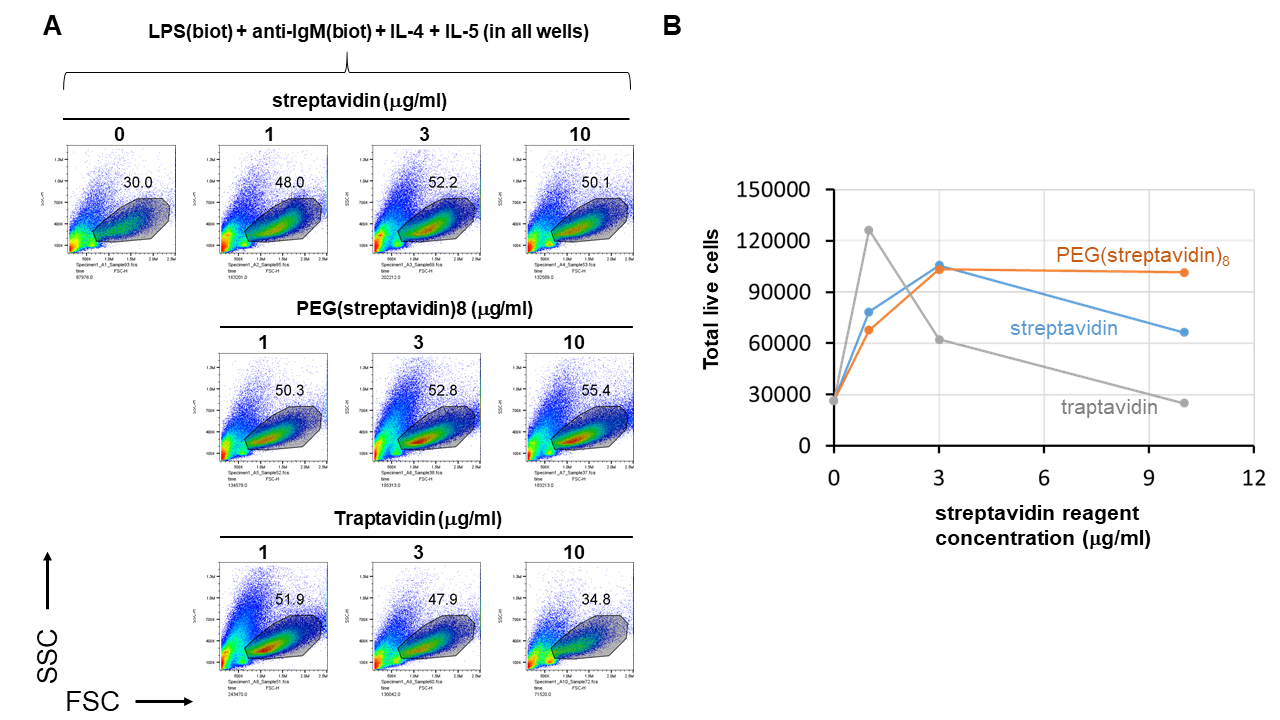


**Suppl. Figure 4. Streptavidin, PEG(streptavidin)_8_ or traptavidin enhance B cell growth.** LPS(biotin) (2 μg/ml) and anti-IgM F(ab’)_2_ (0.3 μg/ml) biotin-LPS were incubated with the indicated amounts of streptavidin, PEG(streptavidin)_8_, or traptavidin in the wells of a V-bottom polypropylene plate for 2 hrs at rt (all wells also contained 50 ng/ml IL-4 and 50 ng/ml IL-5). Each well was then added to the corresponding well of resting murine splenocytes (1.5 x 10^5^ cells/ml in each well) in RPMI-FBS plus BME containing. After 4 days in culture, cells were analyzed by flow cytometry. **(A)** The growth of B cells visualized by the typical forward vs side scattering indicates better growth at intermediate streptavidin:biotin conjugate ratios. (**B**) Quantification of the total number of live cells per well for each of the streptavidin type of reagent concentrations shown in (**A**) above. Note that while cell growth in the streptavidin and traptavidin titrations peak at intermediate (1-3 μg/ml) concentrations of these biotin-crosslinking reagents, PEG(streptavidin)_8_ plateaus at 3-10 μg/ml, possibly since each streptavidin-PEG octamer does not conjugate to other octamers to form larger PEG-streptavidin:biotin multimers.
